# Rapid Free‐Breathing and Automated 2D Shimming of the Lung at 3T

**DOI:** 10.1002/mrm.70238

**Published:** 2026-01-04

**Authors:** Pavlos Panos, Grzegorz Bauman, Oliver Bieri

**Affiliations:** ^1^ Department of Biomedical Engineering University of Basel Allschwil Switzerland; ^2^ Department of Radiology, Division of Radiological Physics University Hospital Basel Basel Switzerland

**Keywords:** active, automated, B0 mapping, bSSFP, deep learning, lung, registration, segmentation, shimming

## Abstract

**Purpose:**

To develop and validate a rapid, fully automated method for shimming of the lung.

**Methods:**

For field mapping of the lung, a custom 2D RF spoiled gradient echo sequence was used offering sub‐millisecond echo times. At 3T, four echo‐shifted coronal images were acquired with a minimum TE of 0.57 ms and an inter‐echo spacing of 0.1 ms. Imaging was performed in free‐breathing using three repetitions with a total scan time of 4.2 s. Subsequently, motion correction, field mapping, lung segmentation, and lung shim currents were calculated inline. Finally, the derived shim currents were saved locally, accessible by any custom sequence for application during scan preparation. The shimming method's performance was compared against the vendor's default settings in five healthy volunteers for functional lung imaging with matrix pencil decomposition using balanced steady‐state free precession (bSSFP).

**Results:**

Field mapping and shimming of the lung was successfully performed in all volunteers and derivation of shim currents took less than 10s. At 3T, subject‐specific shimming reduced the mean frequency offset in the lung by up to 45 Hz and the frequency range (max–min) by up to 180 Hz. Overall, this improved bSSFP signal homogeneity, resulting in more uniform functional images.

**Conclusions:**

This work demonstrates a robust, automated shimming solution for lung imaging at 3T, easily integrable into clinical workflows. The technique significantly enhances image quality and reliability for high‐field, bSSFP‐based pulmonary imaging. At lower field strengths, the method can possibly relax TR constraints, reducing SAR and peripheral nerve stimulation.

## 1 Introduction

Balanced steady‐state free precession (bSSFP) has demonstrated excellent clinical perspectives for structural and functional imaging of the lung [1, 2, 3, 4, 5, 6, 7, 8, 9, 10]. At high magnetic fields, however, bSSFP is limited by its inherent sensitivity to main magnetic field inhomogeneities that may lead to so‐called banding artifacts that appear for off‐resonance related precession frequency close to ±*n*/(2·TR), where n is an odd integer [11, 12]. These artifacts can become particularly prominent for large field of views and in the presence of pronounced susceptibility differences, such as for the lung.

A simple strategy to mitigate bSSFP's off‐resonance sensitivity is to substantially shorten its TR [13]. Minimum TR settings, however, may not only be limited by gradient performance but in practice also by MR safety limits on energy deposition (specific absorption rate) and peripheral nerve stimulation (rapid gradient switching). A possible alternative strategy is to maximize the field homogeneity prior to any measurement using patient‐specific shimming.

Generally, the first step in patient‐specific shimming, or simply “shimming”, is to acquire a 2D or 3D map of the magnetic field homogeneity. This map, commonly referred to as “field map,” indicates local frequency offsets (e.g., in Hz) from the system's center frequency. The retrieved field map is then decomposed into spherical harmonics, which enables patient‐specific shimming by adjusting the respective currents of the linear and, if available, higher‐order spherical harmonic shim coils [14]. For calculation of the shim currents, a dedicated optimization algorithm minimizes the presence of off‐resonances either globally (over the entire volume) or locally (within a user‐specified region‐of‐interest).

All clinical MRI systems include vendor‐provided automatic shimming procedures, typically also offering field mapping sequences that are tailored to imaging specific anatomical regions, such as the brain, the heart, or the abdomen. These automated approaches, however, fail when applied to the lung since its signal decays extremely rapidly. At 3T, for instance, the reported T2* times in the lung tissue lie in the hundreds of microseconds [15]. As a result, conventional vendor‐based methods become ineffective when applied to the lung, as field mapping cannot be performed under these conditions. Moreover, the provided methods do not support the definition of complex shim regions or volumes, such as local shimming adapted to the anatomy of single organs.

In this work, a fully automated 2D active shimming approach for the lung is presented and evaluated at 3T for functional lung imaging with bSSFP using matrix pencil decomposition [16]. Our method performs rapid field mapping in free‐breathing using a multi‐echo RF spoiled gradient‐echo (GRE) sequence adapted to the prominent rapid T2*‐related signal decay observed in the lung tissue. Subsequently, field mapping is performed, and shim currents are derived for the lung tissue only and locally stored. The shim settings are accessible by any sequence and can be set prior to image acquisition; demonstrated for functional lung imaging with bSSFP using matrix pencil decomposition [16] at 3T.

## 2 Methods

All imaging was performed on a clinical 3T whole‐body MRI system (MAGNETOM Prisma, Siemens Healthineers, Forchheim, Germany) using a combination of an 18‐channel thorax array and a 32‐channel spine coil receive array. The system offered second‐order shimming capabilities. The study was approved by the local ethics committee, and written informed consent was obtained from all participants prior to imaging. A total of five healthy volunteers (four female, one male) were examined in a supine position during free‐breathing.

### 2.1 TR‐Shifted Gradient Echoes

For field mapping, multiple echoes have to be acquired but conventional mono‐ or bi‐polar gradient echo trains are too slow to account for the rapid T2*‐related signal decay of the lung. To this end, a TR‐by‐TR based echo‐shifting strategy was implemented. In this approach, one single RF spoiled gradient echo (GRE) is acquired per repetition time (TR), but the echo time (TE) is incremented sequentially over a number *n* of successive TRs while the phase‐encoding gradient is kept constant. This generates a series of *n* “echo‐shifted” images with echo times $TE_{j}=\left(j-1\right)\cdot ∆T+TE_{1}$ (j=1,2,…n), where ∆T refers to a predefined, fixed, inter‐echo spacing and TE_1_ to the minimum echo time. Echo‐shifting was implemented in a custom Cartesian 2D GRE sequence, originally developed for functional lung MRI at 3T [17], offering sub‐millisecond echo times and rapid acquisition under free‐breathing conditions.

### 2.2 Field Mapping

For every voxel position, a set of complex signals ${\hat{s}}_{j}=s_{j}+n_{j}$ can be retrieved from the TR‐shifted multi‐echo GRE sequence, where $n_{j}$ represent complex noise. From this, an incremental phase difference factor can be derived,

(1) $${\hat{s}}_{j1}≔{\hat{s}}_{j}/{\hat{s}}_{1}⟹{\hat{\phi }}_{j1}=\arg\left({\hat{s}}_{j1}\right)$$

from a normalization of the complex signals; e.g., to the signal of the first echo, ${\hat{s}}_{j1}$. This allows a simplified phase evolution analysis, since possible phase wrapping issues along the echo train can be mitigated (as can typically be expected to be present in the individual echo signals).

For field mapping, the cost function

(2) $$\chi^{2}\left(\nu ,\phi_{0}\right)≔\sum_{j\geq 1}{\left|{\hat{\phi }}_{j1}-\phi_{j1}\right|}^{2}$$

was minimized to yield the least‐squares estimators

(3) $$\hat{\nu },{\hat{\phi }}_{0}=\text{argmin}\;\chi^{2}\left(\nu ,\phi_{0}\right)$$

for the voxel's local off‐resonance frequency ν (in Hz) and its local phase offset $\phi_{0}$ from the linear incremental phase difference model (see (1))

(4) $$\phi_{j1}≔\frac{\nu }{2\pi }\left(j-1\right)\cdot ∆\mathrm{TE}+\phi_{0}$$

### 2.3 Lung Segmentation Using Deep Learning

For local shimming, a binary lung mask was generated from the first echo magnitude image using a custom‐trained 2D U‐Net convolutional neural network [18]. The network architecture followed the canonical encoder–decoder structure with symmetric skip connections, residual convolutional blocks for improved feature propagation and gradient stability, as well as a central bottleneck layer incorporating 1024 convolutional filters to extract high‐level semantics.

To minimize annotation bias and improve segmentation accuracy, training was performed using a human‐in‐the‐loop framework. In this iterative process, initial ground truth masks were manually delineated and then refined through alternating prediction–correction cycles using the Napari visualization tool. Each cycle involved network inference, manual error correction, and retraining, thereby gradually enhancing the quality of the training labels.

### 2.4 Derivation of Shim Currents

From the field map, shimming of the lung (defined by the binary lung mask region L) was performed by minimizing the cost function.

(5) $$\chi^{2}\left(c\right)≔\sum_{j\in L}{\left|{\hat{\nu }}_{j}-\nu_{j}\right|}^{2}$$

from the model

(6) $$\nu ≔\frac{\gamma }{2\pi }\left(a\cdot c\right)$$

as generated by *n* spherical harmonic field functions $a≔\left[a_{0},a_{1},\ldots ,a_{n-1}\right]$ with coefficients $c≔{\left[c_{0},c_{1},\ldots ,c_{n-1}\right]}^{T}$.

Since, in this work, scanning was performed in 2D and in coronal orientation, shimming becomes ambiguous with respect to the local frequency offset and magnetic field functions with dependencies in the y‐direction. To resolve this, we treated the y‐position as an arbitrary offset and assigned it entirely to the global frequency term by setting y=0 in the base functions. For zeroth to second order shimming (as available in this work) the nine magnetic field functions are of form [19]:

(7) $$a∼\left\{\begin{array}{c} \begin{array}{l} 1\\ z\\ x \end{array}\\ y\\ z^{2}-\left(x^{2}+y^{2}\right)/2\\ \mathrm{zx}\\ \mathrm{zy}\\ x^{2}-y^{2}\\ \mathrm{xy} \end{array}\right.$$

with coefficients $c≔{\left[c_{0},c_{1},\ldots ,c_{8}\right]}^{T}.$This effectively eliminated the contributions of $c_{\left\{3,6,8\right\}}$ that depend linearly on y, while allowing the remaining coefficients to adjust freely. Thus, for the cost function (5), these terms were excluded from the optimization, and a simple linear least‐square algorithm was used to minimize:

(8) $$\hat{c}=\text{argmin}\;\chi^{2}\left(c\right)$$

to find the local frequency offset ($\gamma /\left(2\pi \right)\cdot c_{0}$) and the remaining first to second order shim currents $c_{\left\{1,2,4,5,7\right\}}$ within the lung tissue only.

### 2.5 Implementation of Automated 2D Shimming

Automated 2D shimming was implemented using the vendor's standard sequence programming (IDEA) and Image Calculation Environment (ICE) together with a recently released framework that allows image data post‐processing using containerized Python modules (OpenRecon).

Two plugins were developed for integration with imaging sequences in the IDEA framework that can be activated in the user interface by:

- A *Calc Shim* flag to perform an inline post‐processing of complex multi‐echo data to generate field maps, to calculate the shim currents and to store them locally in a structured plain‐text shim configuration file, and
- An *Adjust Shim* flag to read the plain‐text shim configuration file and to subsequently set the retrieved shim currents; this action is executed at the end of the sequence preparation routine.

If *Calc Shim* is selected, all data are transferred in a first step from ICE to OpenRecon. In OpenRecon, a non‐rigid image registration [20] is performed between the first echo‐time magnitude image and all other succeeding magnitude images, and the resulting deformation fields are then applied to the corresponding phase images to ensure consistent voxel‐wise phase alignment. Then, a DL‐based lung segmentation is performed on the first echo‐time magnitude image. Subsequently, a field map is computed from the registered phase data, and optimal lung shim currents are derived, as described before. Finally, the estimated lung shim settings are transferred back from OpenRecon to ICE and exported to a locally stored, structured plain‐text shim configuration file.

If *Adjust Shim* is selected, the sequence automatically accesses the shim configuration file before starting the acquisition, parses its parameters, and sets the retrieved shim currents by executing a corresponding command in the Windows command window.

### 2.6 Automated 2D Lung Shimming at 3T

The *Calc Shim* and *Adjust Shim* plugins were added to the proposed custom echo‐shifted GRE sequence. Four coronal images of the chest were acquired with an initial echo time $\left(TE_{1}\right)$ of 570 μs and an inter‐echo spacing of 100 μs. Imaging was performed during free‐breathing with an in‐plane matrix of 128 × 128, a rectangular field of view (FOV) of 450 × 450 mm^2^, and a slice thickness of 12 mm. Additional sequence parameters included a flip angle of 7°, asymmetric echo readout with a bandwidth of 1786 Hz/pixel, parallel imaging using GRAPPA [21] with an acceleration factor of 2 and 24 autocalibration lines. The total acquisition time for the *n* = 4 echo‐shifted images was 1.2 s. Furthermore, the acquisition was repeated three times (covering approximately one full respiratory cycle) to mitigate possible field modulations from the breathing phase by averaging after image registration. The total scan time required for field mapping of the lung was thus less than 4 s.

The *Adjust Shim* option was added to a custom ultra‐fast bSSFP sequence [22] applied for ventilation and perfusion imaging of the lung using matrix pencil decomposition (MP) [16]. Scanning was always performed in coronal orientation with 3.33 images per second. Sequence parameters for the ultra‐fast 2D bSSFP scan were: TR = 1.38 ms, TE = 0.6 ms, flip angle *α* = 25°, field‐of‐view = 450 × 450 mm^2^, bandwidth = 2170 Hz/px, acquisition matrix = 128 × 128 (interpolated to 256 × 256), GRAPPA factor 2. A total of 120 images were acquired per slice in 36 s. MP‐MRI was evaluated for two shim settings in five healthy volunteers: (i) the system's default setting provided by the vendor (“tune‐up”), and (ii) a lung‐specific shimming using the proposed automated shimming using the echo‐shifted GRE method. The functional ventilation‐ and perfusion‐weighted maps were generated using an automated processing pipeline—TrueLung [23].

## 3 Results

The echo‐shifted GRE sequence was tested and evaluated for typical coronal slice locations used for functional lung MRI. The entire process—from image acquisition to the storage of the shim configuration file—took less than 10 s. Although the proposed framework supports iterative shim refinement, a single‐pass correction proved sufficient, as further iterations did not significantly alter the shim settings.

**FIGURE 1:**
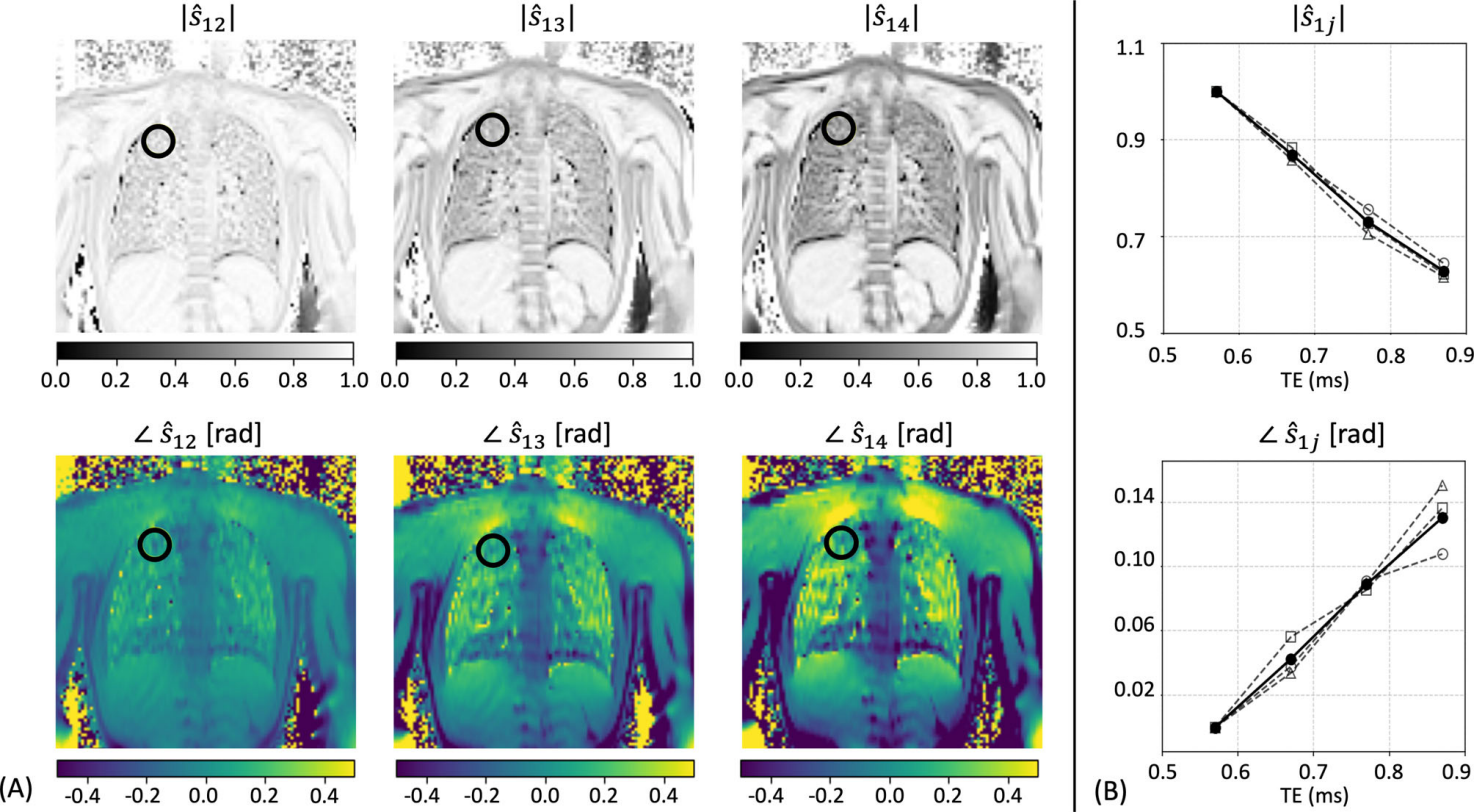
(A) Normalized magnitude and incremental phase images (see Equation 1 in the Methods section) derived from the complex image data acquired using the proposed custom echo‐shifted GRE sequence in a healthy volunteer after co‐registration and averaging over the repetitions (for the base images, see Supporting Information Figure S1). (B) Time evolution of the magnitude and incremental phase for a selected region‐of‐interest in the lung parenchyma (as indicated by the black circle line in A). Shown is the ROI‐averaged time evolution, as observed within each repetition (dashed lines with open circles, triangles, and squares), as well as averaged over the repetitions (solid line, filled circles).

The sub‐millisecond echo times make it possible to yield high‐quality phase images of the lung without noticeable artifacts and degradations (see Supporting Information Figure S1). The corresponding (non‐trivial) normalized magnitude and phase echo‐time images (see Equation 1 in the Methods section) are shown in Figure 1A after co‐registration and averaging over repetitions. A rapid pronounced signal decrease is observed for the lung tissue. Similarly, the phase images reveal a pronounced time evolution, but note that, due to the short echo‐time differences, the incremental phase images are essentially free of any phase wraps.

The time evolution of the normalized signal and of the incremental phase is depicted in Figure 1B for a small selected region‐of‐interest (ROI) within the apex of the lung. For the log‐transformed magnitude data (averaged over the ROI and the repetitions), a simple linear least‐squares fitting (not shown) indicates a T2^*^ as short as 640 μs (*R* ^2^ = 0.998) for the lung parenchyma; in agreement with expectations. The apical lung shows a linear dependency with increasing echo‐times; in agreement with expectations and the presumed phase model (see Equation 4). For the selected ROI, the repetition‐averaged off‐resonance frequency was found to be 70 Hz (*R* ^2^ = 0.999). Generally, averaging over multiple co‐registered measurements helps to mitigate potential instabilities in the lung phase, as expected. Overall, Figure 1 (in combination with the Supporting Information Figure S1) demonstrates that the proposed sequence and method are very well suited for field mapping of the lung at 3T.

**FIGURE 2:**
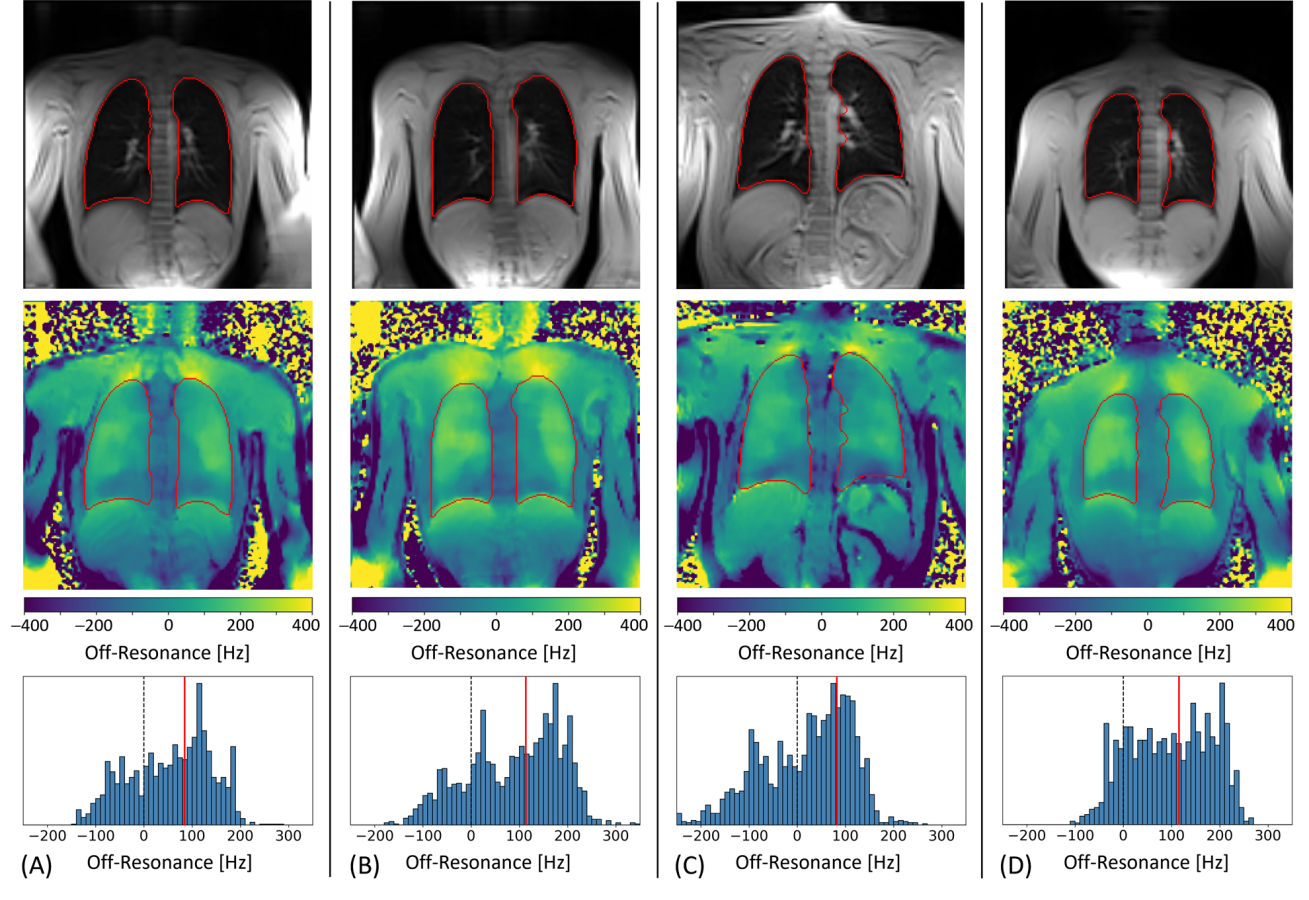
Off‐resonance field maps from four representative healthy volunteers acquired at 3T. The first two rows show: Coronal magnitude images (first row) and the corresponding field maps (second row) with lung contours outlined in red, and the bottom row displays corresponding histograms of off‐resonance values within the lungs. A common pattern of prominent frequency shifts appears near the diaphragm and central airways, but with significant inter‐subject variability. For the slices shown, the mean frequency offsets (red solid lines) are (A) 85 Hz, (B) 112 Hz, (C) 81 Hz, and (D) 107 Hz, with corresponding frequency ranges of 434, 511, 527, and 370 Hz.

The results of the proposed DL‐based lung segmentation method together with the estimated field map are collected exemplarily in Figure 2 for four healthy volunteers. A pronounced off‐resonance variation is observed around the diaphragm, with negative frequency offsets in the lung above the liver, as well as close to the apex of the lung, with strong positive off‐resonances in the region of the cervical pleura. This finding was consistent for all volunteers and at all slice locations (data not shown). While most subjects exhibited a positive average frequency shift within the lung (ranging from −22 to 128 Hz), the histograms varied broadly in shape (with a max‐min frequency range between 350 and 530 Hz).

**FIGURE 3:**
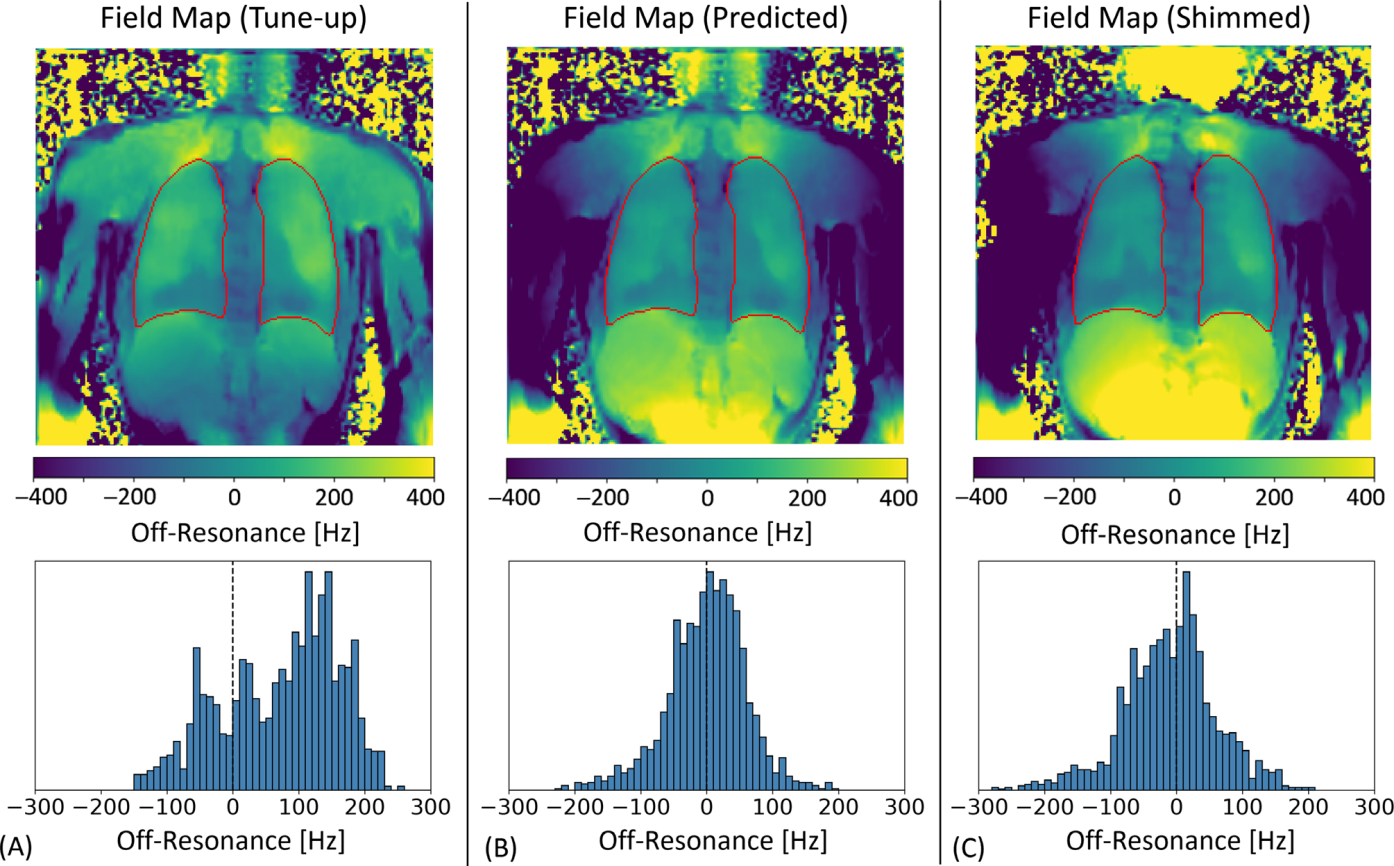
Subject‐specific B_0_ shimming in a representative volunteer at 3T. Top row: Coronal off‐resonance maps under three shim configurations. (A) Default vendor‐provided global shim (“tune‐up”). (B) Simulated field distribution following application of the shim currents predicted by the pipeline, and (C) Measured field map acquired after applying the calculated shim currents on the scanner. Bottom row: Corresponding histograms of off‐resonance values restricted to the segmented lung mask. Notably, the post‐shim map closely matches the prediction, confirming the accuracy of the modeled correction. The histogram demonstrates both narrowing and recentering of the off‐resonance distribution around 10 Hz. Total shimming time, including acquisition and processing, was under 10 s. These results illustrate that single‐pass, subject‐specific correction yields substantial improvements in B_0_ homogeneity without requiring iterative refinement.

Automated 2D lung shimming is demonstrated in Figure 3 for one volunteer. Field maps and off‐resonance histograms are compared for a single coronal slice position using the vendor's default setting (Figure 3A), for the predicted residual field map after optimization of the shim currents (Figure 3B), and for the measured residual field map after applying the correction (Figure 3C). In this example, the mean off‐resonance within the lung was reduced from 54 Hz (in tune‐up) to 11 Hz after shimming, corresponding to a 43 Hz improvement. Across all subjects, the mean lung off‐resonance improved by up to 45 Hz, with reductions in the frequency range by up to 180 Hz. This confirms the effectiveness of the proposed shim calibration in reducing both global frequency offsets and regional inhomogeneities in the lung tissue. Although residual inhomogeneities remained—particularly near the diaphragm—the corresponding histograms revealed a marked centering and narrowing of the lung‐specific off‐resonance distributions.

**FIGURE 4:**
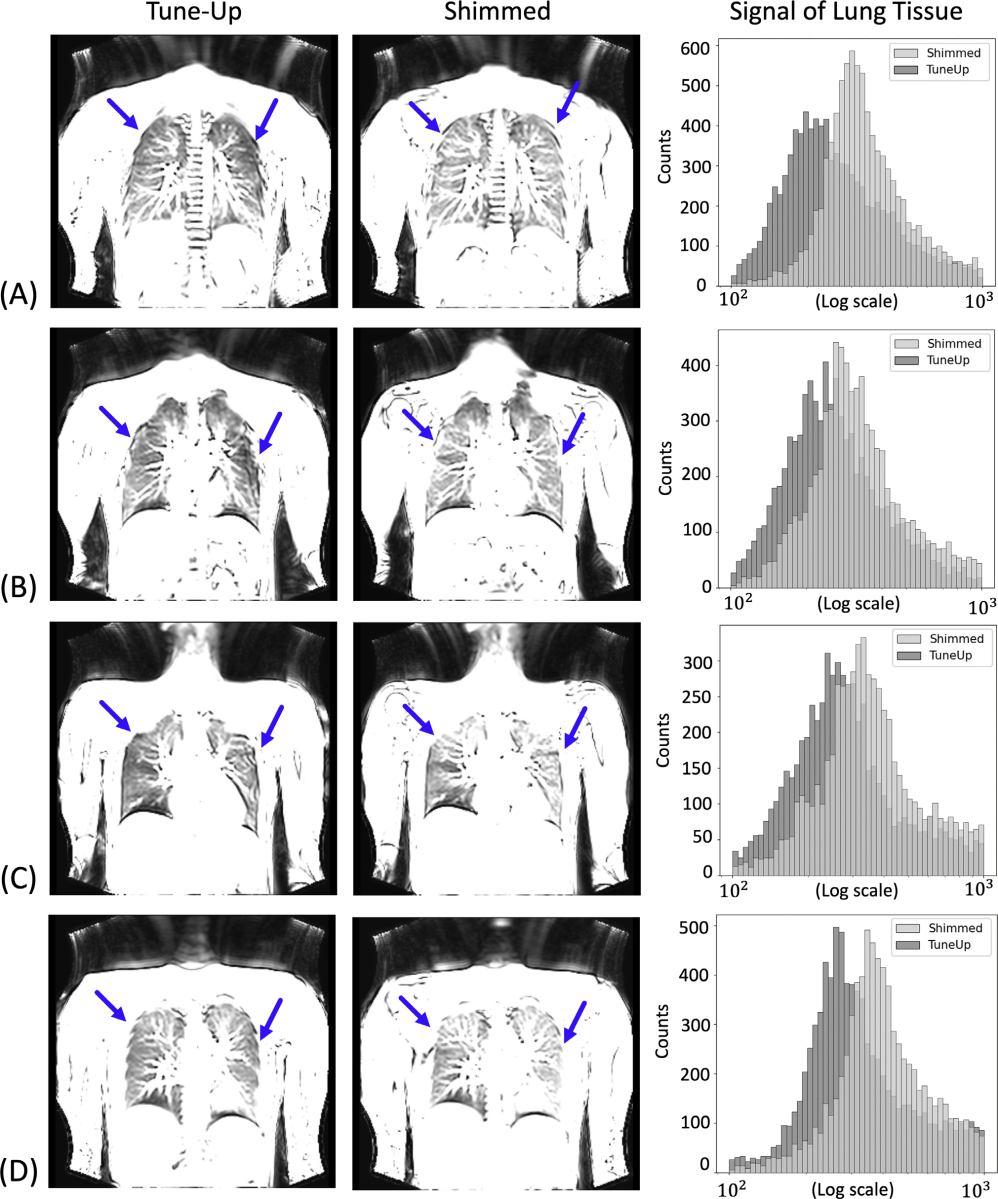
Impact of subject‐specific B_0_ shimming on frequency‐sensitive bSSFP imaging at 3T. Left column displays harshly windowed coronal ultra‐fast bSSFP images acquired using the vendor‐provided default shim configuration (“Tune‐Up”). The middle column shows corresponding slices acquired following the application of subject‐specific shim currents derived from the automated pipeline (“Shimmed”). Substantial improvements are observed in overall signal uniformity and anatomical delineation throughout the lung parenchyma in the shimmed images. Prominent signal voids, particularly in lateral and diaphragmatic regions (indicated by blue arrows), are markedly reduced post‐shimming, highlighting the enhanced signal homogeneity. Although some residual shading remains due to sharp local frequency gradients, the corrected images exhibit a significantly more homogeneous frequency response. The right column compares pixel intensity histograms for the lung for each corresponding image pair (A, B, C, D). The histograms show a clear overall shift to higher values and narrowing of bSSFP signal intensities, thus underlining the prospects of proper shimming.

Possible benefits of an individualized lung shim optimization strategy for mitigation of off‐resonance‐related image degradations with bSSFP are highlighted in Figure 4 for four healthy volunteers. Images are intentionally harshly windowed for visualization of off‐resonance‐related signal modulations in the lung tissue. In “tune‐up” mode, pronounced hypo‐intense signal regions, likely generated by off‐resonances that approach the stop‐band region (i.e., frequencies larger than about ±1/(3TR)), can be detected throughout the lungs, particularly in the posterior and basal regions, an observation consistent with the field maps. As expected, the same regions showed marked signal homogeneity improvements and clearer delineation of pulmonary anatomy following the application of a subject‐specific lung shim setting (for a lobar analysis, see Supporting Information Figure S2). These improvements suggest that functional lung MRI with bSSFP benefits from a rapid 2D automated shimming of the lung at 3T.

**FIGURE 5:**
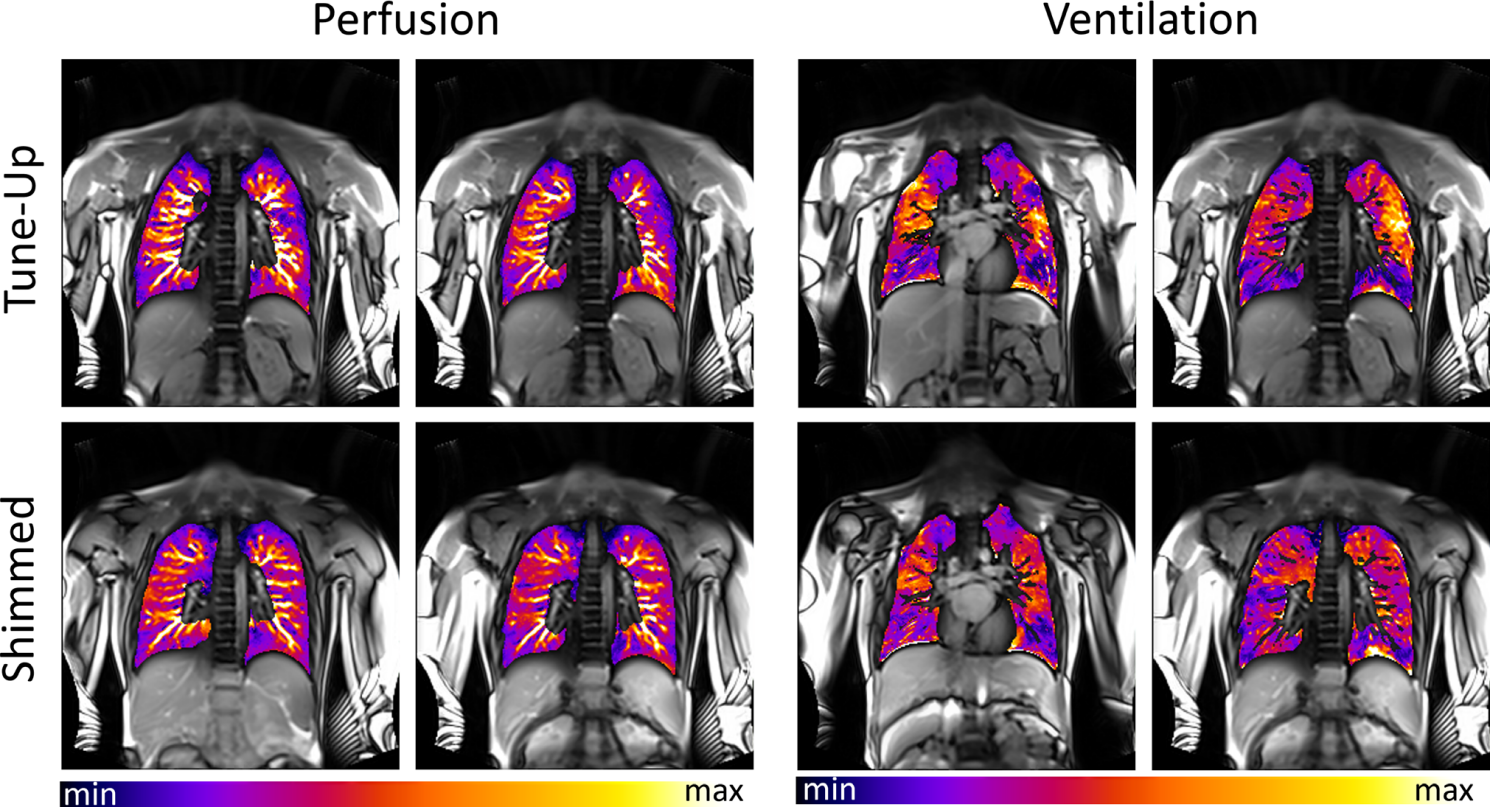
Pulmonary perfusion and ventilation images from MP‐MRI using ultra‐fast bSSFP in healthy volunteers. Functional maps are color‐coded and overlaid on anatomical reference images. Active shimming led to markedly improved homogeneity and signal consistency, particularly in peripheral lung regions and near the diaphragm. Structural features appear more symmetric, and regions previously exhibiting signal dropouts—especially in posterior and basal zones—demonstrate enhanced perfusion and ventilation intensity following shimming. These improvements support the use of individualized shimming for reliable and artifact‐minimized functional lung imaging.

The prospects of a dedicated lung shimming method for MP MRI are highlighted in Figure 5. With default (“tune‐up”) shim settings, functional maps frequently showed diminished spatial fidelity and signal dropouts, particularly in regions of prominent off‐resonances (Figure 2) and signal hypo‐intensities (Figure 4). In contrast, shimming of the lung prior to MP MRI yielded overall improved ventilation and perfusion maps with more homogeneous signal amplitudes across the lung parenchyma consistent with observations in healthy volunteers (see Supporting Information Figure S3).

## 4 Discussion and Conclusion

A central issue addressed in this work is the inadequacy of conventional vendor‐provided field mapping and automated shimming methods for the lung. At high magnetic fields, the extremely short T2* of the lung parenchyma—at 3T, on the order of just several hundred microseconds—precludes a reliable phase estimation using conventional multi‐echo GRE methods; thus, rendering contemporary shimming methods ineffective. To overcome this limitation, we implemented a TR‐based echo‐shifting strategy, which acquires a series of temporally incremented single echoes over a fixed number of TRs with sub‐millisecond spacing. At 3T, four echoes were acquired over a 300 μs window. The resulting field estimation precision, derived from the residual error of the phase evolution fit, was approximately 25 Hz—about one order of magnitude smaller than the typical off‐resonance range exceeding 150 Hz in lung tissue at 3T, a precision sufficient for characterizing these field deviations. Moreover, by restricting the shim optimization to the segmented lung mask, our approach avoided confounding off‐resonance contributions from adjacent tissues like the liver and the cervical pleura.

Overall, this work demonstrates that a dedicated lung shimming method can lead to a substantial improvement in the quality and reliability of frequency‐sensitive lung MRI, as shown for MP MRI at 3T. While this study's 2D approach is effective for single‐slice applications, a key future direction is its extension to a full 3D volumetric shim. A 3D solution would be particularly beneficial for techniques like whole‐lung structural imaging [24]. Applying and validating this rapid shimming technique in patient cohorts with pulmonary diseases is a crucial next step to translate these technical improvements into clinical routine.

In conclusion, the proposed method provides a rapid and robust, fully automated shimming solution for the lung. The integration of ultra‐short echo‐time field mapping with deep learning segmentation in a seamless inline workflow provides substantial improvements in both anatomical and functional MRI of the lung at high field using bSSFP.

## Funding

This work was supported by the Swiss National Science Foundation (320030_219186).

## Conflicts of Interest

The authors declare no conflicts of interest.

## Supporting information


**Figure S1:** Magnitude and phase images as generated by the proposed custom echo‐shifted GRE sequence. The four echo‐time images acquired in each repetition are shown in (A), (B), and (C). The solid orange line in (A) and the dashed orange line in (B) and (C) are used to indicate the position of the diaphragm during the first repetition. This demonstrates the necessity for image registration prior to any averaging.
**Figure S2:** Impact of proper lung shimming on lobar bSSFP signals within the pulmonary parenchyma. (A) Delineation of boundaries for the segmented lobes from a bSSFP image: (i) right upper lobe, (ii) right lower lobe, (iii) left upper lobe, (iv) left lower lobe. (B–E) Corresponding lobar pixel intensity histograms using the vendor‐supplied default shim settings (i.e., “TuneUp”, shown in dark gray) and using the proposed active shimming sequence (i.e., “Shimmed,” shown in light gray). For all segmented regions, active shimming resulted not only in a narrowing of the histograms but also in a shift toward higher mean signal intensity values. This demonstrates that for bSSFP using a dedicated lung shimming can result in a substantial overall signal enhancement and improved signal uniformity; being is a prerequisite for robust functional image analysis.
**Figure S3:** Impact of proper lung shimming on functional images of the lung, as shown in Figure 5. (A, B) The histograms compare the distribution of lung perfusion between predefined shim settings (“tune up”) and after dedicated lung shimming (“shimmed”) for the functional images shown in column one and column two of Figure 5. (C, D) The histograms compare the distribution of fractional lung ventilation between predefined shim settings (“tune up”) and after dedicated lung shimming (“shimmed”) for the functional images shown in column three and four two of Figure 5. Quantitatively, the perfusion maps show a reduction in the standard deviation of 2% (A) and 14% (B). The improvement is even more pronounced in the fractional ventilation maps, where a clear and strong narrowing of the distribution is observed, leading to a substantial STD reduction of 42% (C) and 35% (D). Collectively, these results robustly demonstrate that the proposed active shimming method delivers superior homogeneity and quality in functional lung image data.

## Data Availability

The data that support the findings of this study are available from the corresponding author upon reasonable request.
