# Supplementary material for: Rapid Free‐Breathing and Automated 2D Shimming of the Lung at 3T
Source: Magn Reson Med. 2026 Jan 4;95(5):2737–45. doi: 10.1002/mrm.70238 (PMC12962199; doi:10.1002/mrm.70238)
Supplement: Supplementary file 1 — Figure S1: Magnitude and phase images as generated by the proposed custom echo‐shifted GRE sequence. The four echo‐time images acquired in each repetition are shown in (A), (B), and (C). The solid orange line in (A) and the dashed orange line in (B) and (C) are used to indicate the position of the diaphragm during the first repetition. This demonstrates the necessity for image registration prior to any averaging. Figure S2: Impact of proper lung shimming on lobar bSSFP signals within the pulmonary parenchyma. (A) Delineation of boundaries for the segmented lobes from a bSSFP image: (i) right upper lobe, (ii) right lower lobe, (iii) left upper lobe, (iv) left lower lobe. (B–E) Corresponding lobar pixel intensity histograms using the vendor‐supplied default shim settings (i.e., “TuneUp”, shown in dark gray) and using the proposed active shimming sequence (i.e., “Shimmed,” shown in light gray). For all segmented regions, active shimming resulted not only in a narrowing of the histograms but also in a shift toward higher mean signal intensity values. This demonstrates that for bSSFP using a dedicated lung shimming can result in a substantial overall signal enhancement and improved signal uniformity; being is a prerequisite for robust functional image analysis. Figure S3: Impact of proper lung shimming on functional images of the lung, as shown in Figure 5. (A, B) The histograms compare the distribution of lung perfusion between predefined shim settings (“tune up”) and after dedicated lung shimming (“shimmed”) for the functional images shown in column one and column two of Figure 5. (C, D) The histograms compare the distribution of fractional lung ventilation between predefined shim settings (“tune up”) and after dedicated lung shimming (“shimmed”) for the functional images shown in column three and four two of Figure 5. Quantitatively, the perfusion maps show a reduction in the standard deviation of 2% (A) and 14% (B). The improvement is even more pronounced in the frac [file MRM-95-2737-s001.docx]

**Supporting Information**


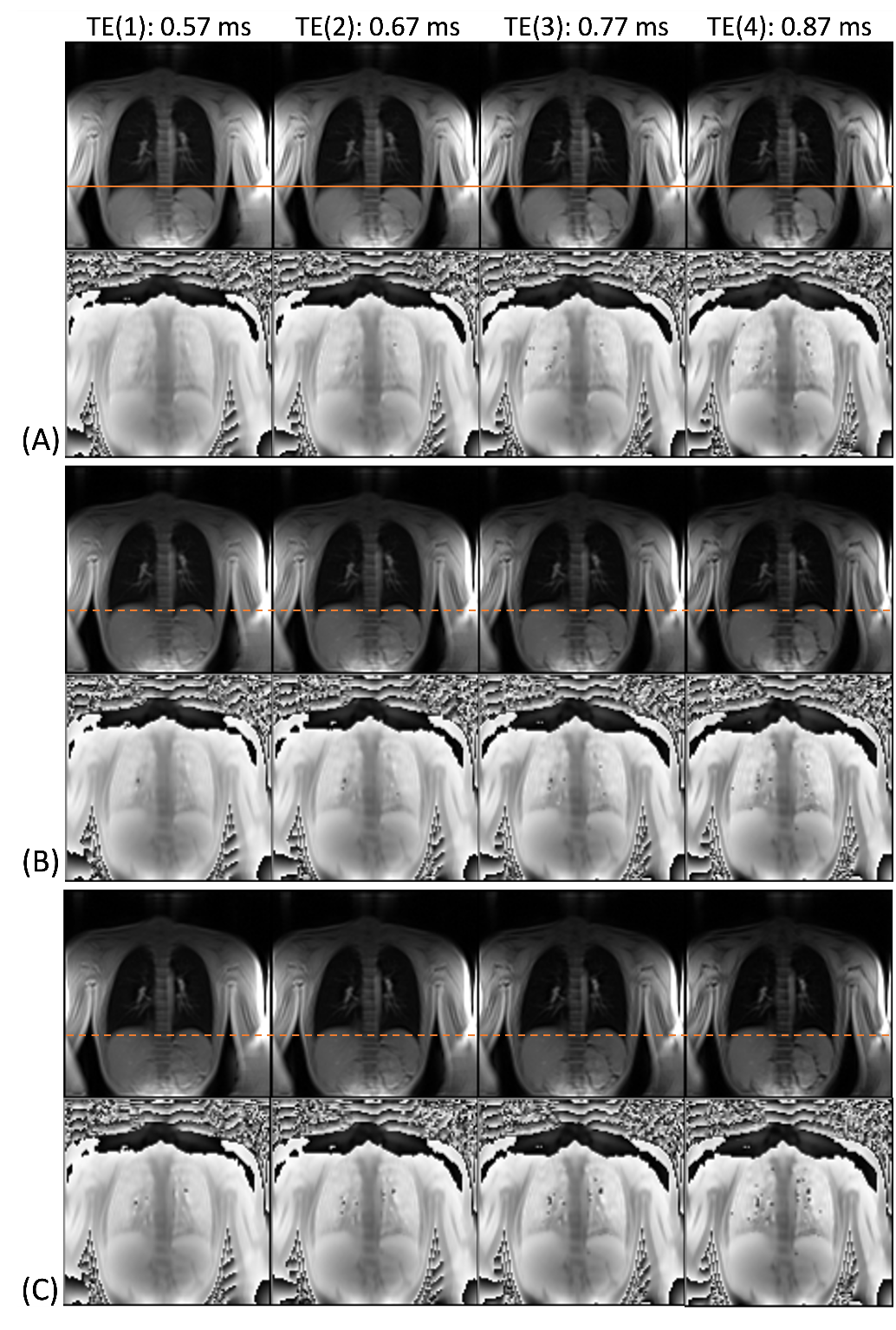


**Figure S1:** Magnitude and phase images as generated by the proposed custom echo-shifted GRE sequence. The four echo-time images acquired in each repetition are shown in (A), (B), and (C). The solid orange line in (A) and the dashed orange line in (B) and (C) are used to indicate the position of the diaphragm during the first repetition. This demonstrates the necessity for image registration prior to any averaging.


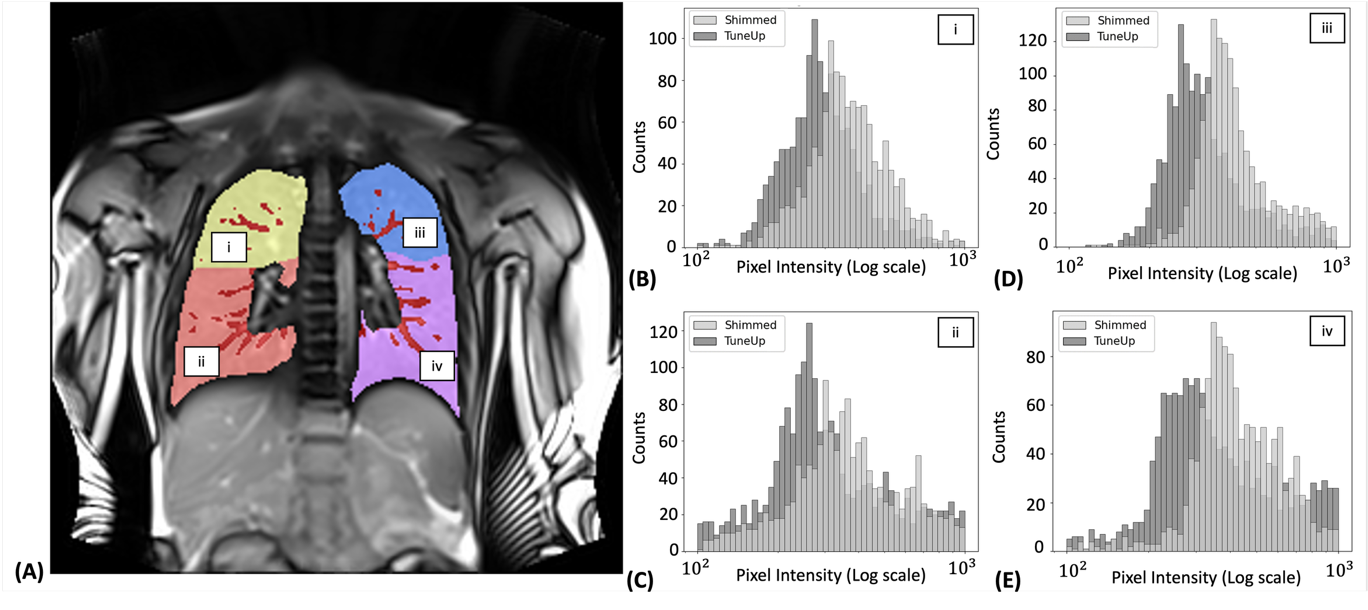


**Figure S2**: Impact of proper lung shimming on lobar bSSFP signals within the pulmonary parenchyma. (A) Delineation of boundaries for the segmented lobes from a bSSFP image: (i) right upper lobe, (ii) right lower lobe, (iii) left upper lobe, (iv) left lower lobe. (B-E) Corresponding lobar pixel intensity histograms using the vendor-supplied default shim settings (i.e., "TuneUp", shown in dark gray) and using the proposed active shimming sequence (i.e., "Shimmed," shown in light gray). For all segmented regions, active shimming resulted not only in a narrowing of the histograms but also in a shift towards higher mean signal intensity values. This demonstrates that for bSSFP using a dedicated lung shimming can result in a substantial overall signal enhancement and improved signal uniformity; being is a prerequisite for robust functional image analysis.


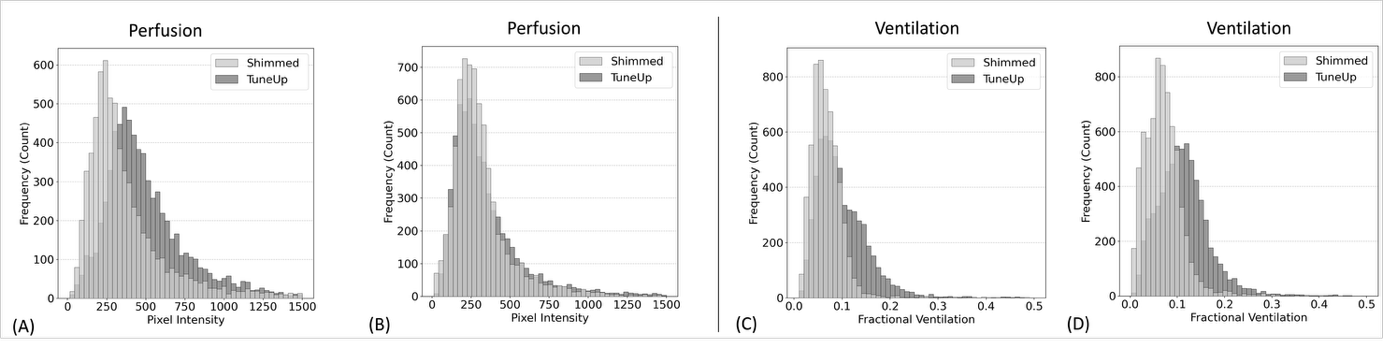


**Figure S3**: Impact of proper lung shimming on functional images of the lung, as shown in Figure 5. (A, B) The histograms compare the distribution of lung perfusion between predefined shim settings (“tune up”) and after dedicated lung shimming (“shimmed”) for the functional images shown in column one and column two of Figure 5. (C, D) The histograms compare the distribution of fractional lung ventilation between predefined shim settings (“tune up”) and after dedicated lung shimming (“shimmed”) for the functional images shown in column three and four two of Figure 5. Quantitatively, the perfusion maps show a reduction in the standard deviation of 2% (A) and 14% (B). The improvement is even more pronounced in the fractional ventilation maps, where a clear and strong narrowing of the distribution is observed, leading to a substantial STD reduction of 42% (C) and 35% (D). Collectively, these results robustly demonstrate that the proposed active shimming method delivers superior homogeneity and quality in functional lung image data.
